# Artificial Intelligence Models for Dysphonia Patient Education

**DOI:** 10.1002/ohn.70030

**Published:** 2025-10-13

**Authors:** Rebecca An Ho, Esha Shah, John Sebastian De Armas, Kenneth Yan, Rachel Kaye

**Affiliations:** ^1^ Department of Otolaryngology Head and Neck Surgery Rutgers New Jersey Medical School Newark New Jersey USA

**Keywords:** artificial intelligence, laryngology, patient education

## Abstract

**Objective:**

Evaluate and compare artificial intelligence (AI) chatbot responses to queries extrapolated from statements by Dysphonia International (DI).

**Study Design:**

Cross‐sectional analysis.

**Setting:**

Online using ChatGPT, Google AI Overview (GAO), and DI.

**Methods:**

Two board‐certified otolaryngologists and an otolaryngology resident each blindly rated ChatGPT, GAO, and DI responses to spasmodic dysphonia, muscle tension dysphonia, and vocal tremor questions. Response comprehensiveness, accuracy, appropriateness to patients, and patient safety were graded on a 5‐point Likert scale. Flesch‐Kincaid Grade Level (FKGL), Flesch Reading Ease Score (FRES), Gunning Fog Index (GFI), and average word count assessed readability. Analysis was performed with one‐way analysis of variance (ANOVA) and Tukey honestly significant difference (HSD); significance was set at *P* < .05.

**Results:**

ChatGPT was significantly more comprehensive than both GAO (*P* < .01) and DI (*P* = .0357). ChatGPT was significantly more accurate than both GAO (*P* < .01) and DI (*P* < .01), while GAO was more accurate than DI (*P* < .01). ChatGPT was found to be significantly more patient‐friendly than both GAO (*P* < .01) and DI (*P* < .01). ChatGPT had significantly safer recommendations than GAO (*P* < .01). ChatGPT had a significantly higher FKGL than GAO (*P* < .01), and a lower FRES than both GAO (*P* < .01) and DI (*P* < .01), indicating a more difficult reading level. All resources exceeded the recommended FKGL and GFI scores as well as the FRES of 90 to 100. Interrater reliability was 0.8.

**Conclusion:**

ChatGPT performed significantly better than GAO and DI regarding comprehensiveness, accuracy, appropriateness, and was an overall safer AI resource to use than GAO. All resources were above the recommended reading grade level. AI models prove to be a formidable alternative resource for patient‐centered clinical information.

The internet has rapidly become a primary resource for health‐related information, with more than 55% of the US population reporting use of online searches for medical queries.^1^ While digital platforms offer unprecedented opportunities for self‐driven health education, studies have shown that online health information—particularly in otolaryngology—is often inaccurate and unclear.^2^, ^3^

In recent years, large language models (LLMs) such as ChatGPT (OpenAI) and Google Gemini (Google) have redefined how users interact with the internet, especially in medicine. These artificial intelligence (AI) models, trained via machine learning on publicly available data, generate human‐like responses to user queries, which have already changed the clinical world in terms of medical accessibility.^4^ ChatGPT, one of the most widely used AI models, has more than 180 million users as of 2024 and can produce comprehensive responses across diverse topics.^5^, ^6^ Google Gemini, a newer AI model, leverages Google's extensive search network and has recently been integrated into Google AI Overview (GAO, May 14, 2024)—summarized search results generated from online Google searches that are designed to provide users with key information from web sources and Google's Knowledge Graph.^7^, ^8^

Although AI platforms have increased access to otolaryngology‐related patient education, their accuracy in answering patient questions about common clinical conditions remains inconsistent.^5^, ^9^, ^10^, ^11^ Similarly, AI models have demonstrated variable performance in answering otolaryngology board examination questions, developing differentials and treatment plans, and adhering to clinical practice guidelines.^12^, ^13^, ^14^, ^15^, ^16^, ^17^ While prior studies have assessed AI performance across otolaryngology subspecialties, research specifically evaluating AI applications in laryngology patient education is lacking.^12^, ^16^, ^17^

Dysphonia, or abnormal voice quality, impairs communication and quality of life in nearly 18 million people in the United States.^18^ It is a common pathology in the elderly population due to vocal bowing/presbyphonia and may arise from other pathologies involving the vocal folds or laryngeal muscles, including neurolaryngological conditions such as spasmodic dysphonia, muscle tension dysphonia (MTD), vocal tremor, vocal cord paralysis/paresis, and respiratory dystonia.^19^ Dysphonia International (DI), a nonprofit organization advised by a multidisciplinary team of researchers, laryngologists, and speech pathologists, provides education and support for patients with these disorders. DI was founded more than 30 years ago and has served globally as a baseline resource for physicians and patients alike to learn more about dysphonic conditions.^20^

Despite the growing literature on AI in otolaryngology, studies focusing on laryngological disorders remain limited. Furthermore, while ChatGPT and Google Gemini have been compared, there are no studies evaluating GAO, a potentially more accessible version of Google Gemini. This study aims to assess the utility of ChatGPT and GAO in answering common patient questions about spasmodic dysphonia, MTD, and vocal tremor, and comparing their responses to patient information provided by DI.

## Methods

### Data Collection

DI was chosen as the gold standard for this study as it is a publicly available 501(c)3 organization that is one of the only epicenters for research, patient education, and support for disorders involving dysphonia.^20^ DI was accessed on August 10, 2024, and patient information for three different pathologies was accessed under “Voice Conditions”: spasmodic dysphonia, MTD, and vocal tremor. Each of these pathologies has its own webpage and is divided into subsections about the pathology, such as “Who is affected by tremor?” within the vocal tremor webpage. While some subsections were titled as questions, some were titled as statements such as “Symptoms of Vocal Tremor.” The available pathologies of vocal cord paralysis and respiratory dystonia, although available on the DI platform, were not included as vocal cord paralysis had overlapping information with that of vocal tremor, and the webpage of respiratory dystonia did not have any subsections. The three pathologies and their associated generated questions were downloaded onto a Microsoft Word (V.16.86, Microsoft Corporation) document for a total of 16 questions (Table 1). For subsections that were not phrased as questions, the authors converted statements into the form of questions, for example “Causes of Spasmodic Dysphonia” subsection was converted into the question, “What are the causes of spasmodic dysphonia?” This nonhuman subject research was approved by the Rutgers New Jersey Medical School Institutional Review Board (Pro2025001148).

**Table 1:** Dysphonia Queries Input Into Artificial Intelligence (AI) Models, Extracted From Dysphonia International (DI)

Topic	Question
Spasmodic dysphonia	What are the symptoms of spasmodic dysphonia?^a^
	Who gets spasmodic dysphonia?
	How is spasmodic dysphonia diagnosed?^a^
	What is the cause of spasmodic dysphonia?^a^
	What is the treatment for spasmodic dysphonia?^a^
Muscle tension dysphonia	Who gets muscle tension dysphonia?
	What are the symptoms of muscle tension dysphonia?^a^
	How is muscle tension dysphonia diagnosed?^a^
	What are the causes of muscle tension dysphonia?^a^
	What is the treatment for muscle tension dysphonia?^a^
	What is the difference between muscle tension dysphonia and spasmodic dysphonia?^a^
Vocal tremor	What is the difference between voice tremor and spasmodic dysphonia?^a^
	Who is affected by vocal tremor?
	What are the symptoms of vocal tremor?^a^
	How is vocal tremor diagnosed?^a^
	What is the treatment for vocal tremor?^a^

^a^ Statement that was converted to question.

### AI Response Generation

On August 13, 2024, all 16 questions were individually queried to the most recent version of ChatGPT‐4 “omni” (ChatGPT‐4o, released August 8, 2024) with a “new chat” to avoid bias and influence on subsequent questions within the same chat. Responses were copied and pasted into the Word document under their respective questions, labeled as “Response A.” All 16 questions were then separately queried via their own “Incognito Window” in Google Chrome (Version 131.0.6778.265). The response under the “AI Overview” that was generated was copied and pasted into the same Word document under their respective questions, labeled as “Response B.” All text formatting was removed from these sources so as to not distinguish responses based on appearance. Finally, the respective DI response from the website for each question was copied and pasted and labeled as “Response C.”

### Physician Assessment

Beneath the response (A, B, or C) for each question on the Word document, the following Likert scales were inputted: (1) This response was accurate, (2) This response was comprehensive, (3) This response was appropriate for patient‐level information, and (4) This response provided safe, non‐dangerous information to the patient. The Likert scale was established as 1 (strongly disagree), 2 (disagree), 3 (neutral), 4 (agree), and 5 (strongly agree).

Responses were graded by two board‐certified otolaryngologists, both of whom had completed laryngology fellowships (R.K., K.Y.), as well as an otolaryngology resident (J.S.D.A.). Each physician had their own version of the original Word document so as to not be influenced by each other's responses. All physicians were blinded to what response A, B, or C correlated with in regards to AI models or DI responses. After each physician completed their gradings, results were input into a Microsoft Excel (Version 16.78.3, Microsoft Corporation) file for analysis.

### Readability Assessment

Responses from both AI models and DI were separately graded by two authors (R.A.H., E.S.) via Flesch Reading Ease Score (FRES), Flesch Kincaid Grade Level (FKGL), Gunning Fog Index (GFI), and average word count. FRES is a score measured on a scale of 0 to 100 that measures the reading difficulty of a text based on word count and syllables, with higher scores corresponding to easier readability. An FRES of above 80 correlates with a sixth‐grade reading level. FKGL is also measured based on word count and syllables but is scored on a 0 to 18 scale. Each FKGL score correlates with a specific grade level, with numbers 0 to 12 correlated with their respective grade levels and numbers above 12 with college and graduate levels. GFI is another linguistic measure that focuses on word complexity and sentence length and is measured on a scale from 6 to 17 where, similar to FKGL, each number correlates with grade level.^21^ The American Medical Association (AMA) recommends that all patient education materials be written at a sixth‐grade reading level.^22^

### Statistical Analysis

The tool Character Calculator was used to generate FKGL, FRES, and GFI scores. Descriptive statistical analysis was performed using Microsoft Excel. Analysis of variance (ANOVA) and post‐Tukey analysis were performed using Microsoft Excel via XLMiner Analysis Toolpak.

## Results

### Physician Scores

**Figure 1:**
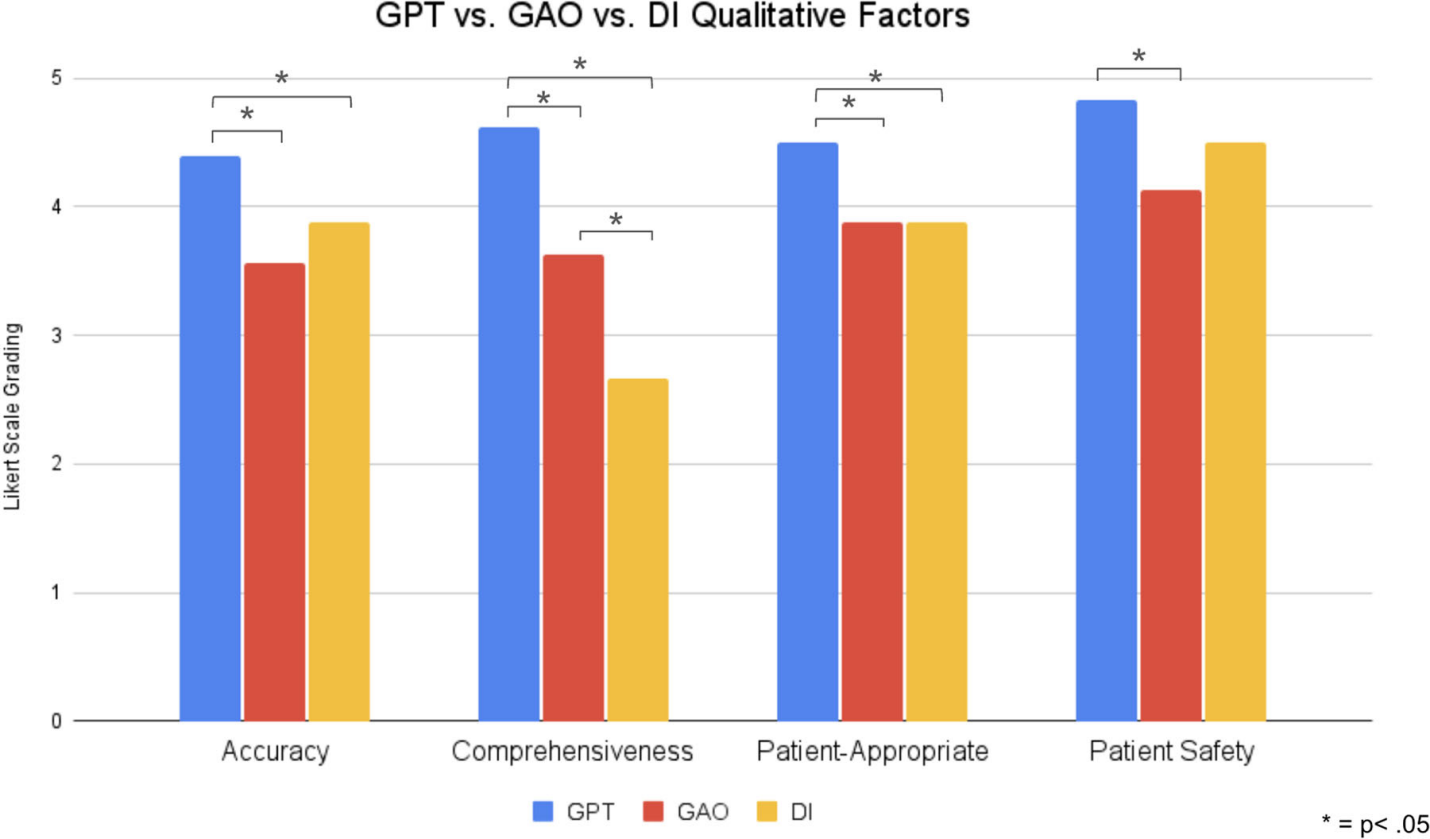
Average physician assessment of ChatGPT versus Google Artificial Intelligence Overview (GAO) versus Dysphonia International (DI) responses.

ChatGPT exceeded both GAO and DI in all aspects according to physician gradings. In regards to the accuracy of information, ChatGPT was significantly more accurate than both GAO (*P* < .001) and DI (*P* = .036). ChatGPT was significantly more comprehensive than both GAO (*P* < .001) and DI (*P* < .001), and GAO was also significantly more comprehensive than DI (*P* < .001). ChatGPT had significantly more appropriate patient material than both GAO and DI (*P* = .00148), and GAO and DI had similar scores. ChatGPT provided significantly safer patient information and recommendations than GAO (*P* < .001). Its safety score was higher than that of DI, but this difference did not meet statistical significance (*P* = .11059) (Table 2, Figure 1).

**Table 2:** Average Qualitative Scores of ChatGPT Versus Google Artificial Intelligence Overview (GAO) Versus Dysphonia International (DI) Responses

Metric	ChatGPT	GAO	DI	Pairwise comparisons
Accuracy	4.40 ± 0.74	3.56 ± 1.00	3.88 ± 1.27	GPT versus GAO: *P *<.001
				GPT versus DI: *P* = .03571
				GAO versus DI: *P* = .29322
Comprehensiveness	4.63 ± 0.57	3.63 ± 1.00	2.67 ± 1.2	GPT versus GAO: *P* < .001
				GPT versus DI: *P* < .001
				GAO versus DI: *P* < .001
Patient‐appropriate	4.50 ± 0.62	3.88 ± 0.84	3.88 ± 1.06	GPT versus GAO: *P* = .00148
				GPT versus DI: *P* = .00148
				GAO versus DI: N/A
Patient safety	4.83 ± 0.56	4.13 ± 1.08	4.50 ± 0.68	GPT versus GAO: *P* < .001
				GPT versus DI: *P* = .11059
				GAO versus DI: *P* = .06262

### Readability Scores

**Figure 2:**
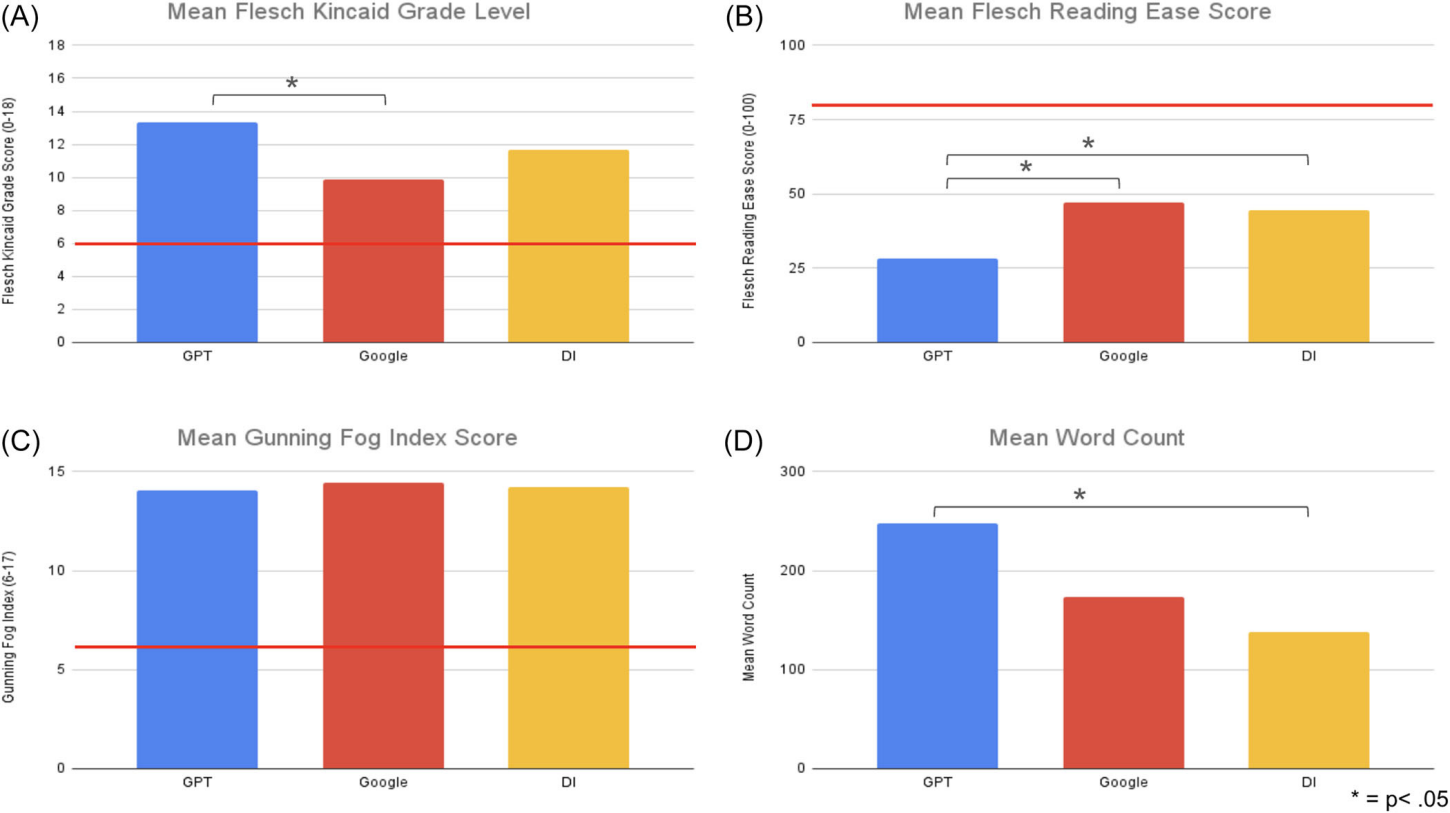
Readability scores by model using (A) Flesch Kincaid Grade Level (FKGL), (B) Flesch Reading Ease (FRES), (C) Gunning Fog Index (GFI), and (D) word count. Scales: FKGL (0‐18), FRES (0‐100), and GFI (6‐17). DI, Dysphonia International.

ChatGPT had both a significantly lower FRES (*P* < .001) and higher FKGL (*P* < .001) compared to GAO, both scores correlating with more difficult readability levels. While GAO had a higher FRES (47.21 ± 9.13 vs 44.34 ± 10.86) and lower FKGL (9.89 ± 1.86 vs 11.64 ± 2.01) than DI, these scores were not significantly different. The GFI for all resources had no significant differences. ChatGPT has a significantly higher average word count than DI (*P* = .00477) (Table 3, Figure 2). Notably, the average FRES, FKGL, and GFI for all reading material generated by AI models and information from DI had responses all above the level of a sixth‐grader, the recommended level for a patient in the United States by the AMA, indicating poor readability for the average patient.

**Table 3:** Average Readability Scales of ChatGPT Versus Google Artificial Intelligence Overview (GAO) Versus Dysphonia International (DI) Responses

Metric	ChatGPT	GAO	DI	Pairwise comparisons
FKGL	13.31 ± 2.26	9.89 ± 1.86	11.64 ± 2.01	GPT versus GAO: *P* < .001
				GPT versus DI: *P* = .06709
				GAO versus DI: *P* = .05116
FRES	28.22 ± 11.64	47.21 ± 9.13	44.34 ± 10.86	GPT versus GAO: *P* < .001
				GPT versus DI: *P* < .001
				GAO versus DI: *P* = .72661
GFI	14.05 ± 2.03	14.46 ± 2.14	14.22 ± 2.47	GPT versus GAO: *P* = .85898
				GPT versus DI: *P* = .97421
				GAO versus DI: *P* = .94899
Word count	248.19 ± 87.85	173.69 ± 43.24	137.94 ± 128.88	GPT versus GAO: *P* = .07290
				GPT versus DI: *P* = .00477
				GAO versus DI: *P* = .52999

Abbreviations: FKGL, Flesch‐Kincaid Grade Level; FRES, Flesch Reading Ease Score; GFI, Gunning Fog Index.

## Discussion

Dysphonia describes a range of abnormal voice conditions and qualities, including changes in pitch, volume, and/or increased efforts to phonate. Neurological etiologies are common and can range from the laryngeal spasms experienced in spasmodic dysphonia to the vocal strain experienced in MTD. In these disorders, dysphonia affects one's ability to communicate, directly impacting psychosocial function with frustration, changes in self‐identity, and vocational issues.^23^ While investigations into the utility of AI in otolaryngology have been well sought out and evolving, literature remains lacking in regards to laryngology‐related topics. This may be in part due to a lack of consolidated laryngology patient materials and education materials regarding common pathologies. With as many as 55% of US adults reporting utilizing the internet for medical information, we find investigations into the quality of AI material regarding dysphonia conditions to be important for those who have problems communicating with their voice.^1^

Although DI was designated the gold standard for this study, we found ChatGPT to perform at a significantly higher level than both DI and GAO on almost all qualitative measures. However, its readability levels were significantly more difficult to read than its counterparts. Notably, all models on average had readability levels above the recommended sixth‐grade level by the AMA. These findings are generally in accordance with otolaryngological AI research comparing ChatGPT and Gemini; several studies found ChatGPT superior to Gemini on qualitative levels including accuracy, comprehensiveness, and adherence to guidelines.^24^, ^25^, ^26^, ^27^, ^28^ Papuc and Scheffler also found ChatGPT to have worse readability measures than Gemini, and this is in agreement with most current literature comparing the readability of these models.^26^, ^29^, ^30^, ^31^ These findings paint the broad picture that although ChatGPT excels in the quality and completeness of its answers, it is often much more difficult to read than its AI counterparts. Moreover, the average word count of ChatGPT in this study is significantly higher than that of DI and nearly that of GAO. This should be taken into consideration regarding the qualitative measures of ChatGPT—though longer responses do not necessarily correlate to better responses, it certainly gives ChatGPT the opportunity to address more aspects of a topic. However, this is not to negate the fact that ChatGPT was deemed more patient‐appropriate and provided less hazardous information than GAO. As GAO is a new feature of Google searches, with its existence being no more than a year old, patients should take this information into account when reading this material. This is important in regards to the more readily available presence of GAO, as the average user has to only search a question within Google to get a GAO response, in contrast to ChatGPT, where one has to specifically seek out this resource.

Several studies in other otolaryngology subspecialties have been conducted evaluating ChatGPT in a similar manner with varying results. In one study, Fazilat et al found ChatGPT responses to be comparable on all levels to an academic rhinological resource, including comprehensiveness, accuracy, readability, and quality.^32^ On the other hand, a study by Gorris et al found ChatGPT to only be accurate and satisfactory half of the time regarding patient queries about thyroid cancer.^33^ Meanwhile, Albehairi et al's study found ChatGPT to perform perfectly in regards to accuracy and clarity, and added that such a chatbot will be helpful for those in remote or resource‐limited areas.^34^ Our study revealed that while these resources are easy to access and appear to perform better than what is currently available on an academic site such as DI, the performance of these bots remains below 100% in all aspects. Though Albehairi et al's point remains true that such resources can help those with limited healthcare access to gain needed medical information, the quality of information generated by AI bots ultimately cannot replace that of a treating physician.

Many otolaryngology papers studying AI ultimately assess if these models can be trusted on their own in regards to patient education and guidance. From the results of this study and other otolaryngological literature investigating these bots, the general consensus appears that while these models can provide supplemental help for patient education, patients still should not rely on them for their medical care.^1^, ^32^, ^33^, ^34^ The varying levels of accuracy, consistency, and readability of the information from AI models provide concern about patient safety. Furthermore, as the information is restricted to what is publicly available, the lack of access to peer‐reviewed medical journals brings to the forefront concerns in regards to the medical resources they are drawing from. Not to mention, ChatGPT‐4o is the most recent publicly available version, and its information only is updated to October 2023. This study remains the first in otolaryngology literature to study AI's performance in laryngology; while ChatGPT outperformed both GAO and DI, we bring to light the comparison of the quality versus quantity of its answers.

The limitations of this study include aspects of the generation of this information and the grading. Our focus on laryngology and a relatively small data set of 16 questions for three pathologies is not fully generalizable to AI's capability within laryngology or other otolaryngology subspecialties. Given that AI models are constantly evolving, updating, and learning from user inputs, it is possible that answers received in this study could vary from answers to the same questions asked at a different time point. That is to say, we interacted with only one version of each chatbot and the information it had at that time—its future responses may differ. Additionally, there are key distinctions between our interactions with the chatbots and the layperson's interactions: most people utilize more than one query per chat with AI, allowing the bot to further elaborate and clarify as the user requires. Questions from patients may also be phrased differently than questions derived from DI, our gold standard for study purposes. As such, the results of this study may be more applicable for general practitioners or resident trainees. Future studies could consider recruiting real patients for question generation and comparison to physician‐generated responses. Furthermore, our study is limited by the lack of a standardized evaluation tool for AI‐generated patient education materials in laryngology. The physician's assessments of the chatbots' responses could be biased by the evaluators' high health literacy. On a similar note, though blinded, the physicians were presented with responses from ChatGPT as Response A, GAO as Response B, and DI as Response C. The consistency in presentation of this data may have influenced reviewers' evaluations, as patterns could have been detected between chatbots' responses across queries. Although the study offers insightful information about how well AI chatbots perform in answering medical questions, these limitations draw attention to the necessity for ongoing research and evaluation as AI technologies advance and are integrated into healthcare procedures and protocols.

## Conclusion

Online AI query platforms have been rapidly increasing in popularity worldwide for a wide range of uses. This study demonstrates the power of these AI models in improving patient understanding of dysphonia and its related pathologies. ChatGPT outperformed GAO and DI in most aspects, with higher scores in accuracy, comprehensiveness, appropriateness, and safety—making it a valuable tool for laryngology patient education. However, as demonstrated by this study, AI models can often require a higher health literacy to fully utilize. Thus, ChatGPT and GAO must be responsibly integrated into patient education under the supervision of board‐certified professionals to maximize their potential in improving medical knowledge and bridging communication gaps between patient and provider.

## Author Contributions

**Rebecca An Ho**, conceptualization, methodology, validation, formal analysis, investigation, resources, data curation, writing—original draft, visualization; **Esha Shah**, formal analysis, data curation, writing—original draft; **John Sebastian De Armas**, data curation, writing—review and editing, supervision; **Kenneth Yan**, writing—review and editing, supervision, project administration; **Rachel Kaye**, conceptualization, writing—review and editing, supervision, project administration.

## Disclosures

### Competing interests

The authors declare no conflicts of interest.

### Funding source

This research received no specific grant from any funding agency in the public, commercial, or not‐for‐profit sectors.
